# Political attitude change over time following COVID-19 lockdown: Rallying effects and differences between left and right voters

**DOI:** 10.3389/fpsyg.2022.1041957

**Published:** 2022-12-15

**Authors:** Nicole Satherley, Elena Zubielevitch, Lara M. Greaves, Fiona Kate Barlow, Danny Osborne, Chris G. Sibley

**Affiliations:** ^1^School of Psychology, The University of Auckland, Auckland, New Zealand; ^2^UQ Business School, The University of Queensland, Brisbane, QLD, Australia; ^3^School of Social Sciences, The University of Auckland, Auckland, New Zealand; ^4^School of Psychology, The University of Queensland, Brisbane, QLD, Australia

**Keywords:** COVID-19, political attitudes, attitude change, rally around the flag, partisanship, regression discontinuity, political ideology

## Abstract

Restrictions to curb the spread of COVID-19 have required widespread compliance over long periods, but citizens’ attitudes to these often change over time. Here, we examine the time course of political attitudes in New Zealand over the months before and after the announcement of the country’s first nationwide COVID-19 lockdown in 2020 using a large-scale national survey (*Ns* = 41,831-42,663). Government satisfaction increased immediately following the lockdown announcement and remained elevated 5 months later. Trust in institutions and political efficacy also increased gradually over the same period. However, these trends varied by political party vote: Compared to center-left voters who supported the largest governing party, center-right voters who supported the opposition party returned to baseline levels of government satisfaction quicker and showed more pronounced dips in their satisfaction with the economy. These same attitudes also predicted compliance with COVID-19 guidelines. Results illustrate a rally-around-the-flag effect during the pandemic and suggest that support wanes faster among center-right (opposition party) voters.

## Introduction

During the COVID-19 pandemic, many nations have witnessed their citizens “rally-around-the-flag” (Mueller, 1973), whereby approval of incumbent political leaders soars in the face of a collective threat (Sibley et al., 2020; Yam et al., 2020; Bol et al., 2021; Lupu and Zechmeister, 2021; Roccato et al., 2021). Such rapid increases in institutional support during times of crisis are thought to be adaptive because they can induce a unified identity among the public which enables a more cohesive response to threat (see Bavel et al., 2020). Despite the impressive body of work documenting this phenomenon, the *duration* of rally effects—both in general and as they relate to COVID-19—is relatively underexplored. This oversight is problematic because unlike some crises (e.g., an earthquake), the COVID-19 pandemic has required prolonged periods of compliance with mitigation efforts, rendering concerns about fatiguing the public with lengthy restrictions on their individual freedoms (World Health Organization, 2020). Clearly, this unique set of factors necessitates tracking attitudes over extended periods to understand how long favorable opinions of political incumbents endure.

Further complicating our understanding of rally effects in the context of COVID-19 is that their magnitude and longevity may vary across the political spectrum, as conservatives and liberals have had contrasting responses to the pandemic. For example, political conservatives (relative to liberals) have been less compliant with health guidelines on social distancing (Becher et al., 2021; Koetke et al., 2021), less likely to engage in precautionary behaviors (Samore et al., 2021), and express less concern about the pandemic in general (Gadarian et al., 2021; Ruisch et al., 2021). Indeed, Shino and Binder (2020) showed that partisanship dampened rally effects among Florida voters in the United States after the state’s Republican governor launched a “Safer at Home” campaign. Likewise, a multi-wave study by Kritzinger et al. (2021) suggests political polarization may help to explain the lack of a rally effect in France at the beginning of the pandemic. Together, these findings suggest that political party affiliation may be a key qualifier to the persistence of any rally effects during the COVID-19 pandemic.

The aims of the current study are twofold: to examine the duration of rally effects in the population and to investigate whether these trends differ by political party voters. We conduct a day-by-day time course analysis of political attitudes in the months prior to—and following—the announcement of New Zealand’s first nationwide lockdown in March 2020. New Zealand’s first nationwide lockdown was remarkable relative to other countries’ pandemic mitigation approaches with respect to its intensity and scope, and has been described as some of the most severe restrictions worldwide (Jones, 2020). Our analyses allow us to identify both how much political attitudes changed—and for how long—in response to the lockdown, and whether these attitudinal trajectories differ between voters for the largest, center-left governing party (Labour) and voters for the largest, center-right opposition party (National). Given the important health implications of compliance during the pandemic, we also examine whether these political attitudes predict willingness to comply with directives from the governing health body in New Zealand.

### Attitude change in response to COVID-19

Crisis points can elicit rally-around-the-flag effects, which refer to a dramatic uptick in support for political leaders and institutions that occupy power (Mueller, 1973). To date, research on rally effects have largely focused on responses to terrorism, with cross-national research showing that support for incumbent governments increases among the public in the wake of an attack (e.g., Chanley, 2002; Satherley et al., 2021) even among opposition party elites (Chowanietz, 2011). Although a distinct threat from terrorism, rally effects have also been documented in response to the COVID-19 pandemic. For example, Baekgaard et al. (2020) showed that even those most vulnerable to economic shocks caused by COVID-19 (i.e., unemployed people) expressed greater trust in political institutions during the initial 3 weeks of Denmark’s lockdown in March 2020. Likewise, Sibley et al. (2020) identified increased satisfaction with, and trust in, the government during the first 18 days of New Zealand’s nationwide lockdown in March 2020 (see also Goldfinch et al., 2021). Further, Bol et al. (2021) demonstrated greater vote intentions for the governing party, trust in government, and satisfaction with democracy across several European countries—although the authors argue this likely resulted from people evaluating lockdown policies as good and necessary.

Although research demonstrates that the COVID-19 pandemic elicited substantial increases in government support, the *longevity* of these effects is poorly understood. This poses a barrier to pandemic mitigation, given the tension between the theorized benefits of rally effects to manage threats (Bavel et al., 2020) and the enduring nature of the COVID-19 pandemic (World Health Organization, 2020). Prior work analyzing political attitudes following various terror events reveals that support for, and satisfaction with, the government can remain elevated for some time, ranging from 8 to 20 weeks (Geys and Hernæs, 2021; Satherley et al., 2021). With respect to the duration of rally effects during *pandemics*, Baekgaard et al. (2020) found increased trust in Denmark persisted 3 weeks after the first COVID-19 lockdown but they did not assess attitudes beyond that point. Bangerter et al. (2012) examined trust in Swiss institutions’ response to the 2009 H1N1/H5N1 pandemic, finding high trust in 2009 but widespread decreases 1 year later. Likewise, Johansson et al. (2021) found that government approval in Sweden declined between April and September 2020 after an initial spike in support. Collectively, this work indicates that rally effects are important for pandemic management, but that they wane over time.

Why rally effects decline over time may be due to the perceived trade-offs caused by restrictions such as lockdowns, border controls, and gathering limits. These strategies reduce illness and death, but can also place heavy burdens on individual freedoms and the economy (e.g., Daniele et al., 2020; Carrieri et al., 2021; Oana et al., 2021). Crucially, these perceived trade-offs change as the pandemic progresses. For example, Naumann et al. (2020) found that German respondents thought the societal benefits of COVID-19 mandates outweighed the economic cost initially, but these perceptions started to decline 2 weeks into Germany’s March 2020 lockdown. Martinez-Garcia et al. (2021) also found lockdown fatigue in relation to economic and psychological impacts in Spain. Specifically, respondents were less willing to be locked down for a long period of time further into the pandemic (i.e., in September 2020 compared to in April 2020). Moreover, as the pandemic wore on, the economic impact of the lockdown had a larger effect than the psychological impact on willingness to comply. Notably, Sibley et al. (2020) identified little-to-no evidence of changes in mean-levels of New Zealanders’ sense of national wellbeing (including satisfaction with business and the economy) within the first 18 days of lockdown (compared to mean levels prior to COVID-19). However, this research was conducted over a short interval, whereas decreases in national wellbeing seem more likely to appear over a greater period.

### Political differences in COVID-19 attitudes

In addition to examining the duration of rally effects, a comprehensive understanding of responses to the pandemic requires a focus on how different people reacted to the various mitigation measures. To these ends, voter differences in COVID-19 attitudes have been widely identified (e.g., Collins et al., 2021), and the magnitude and duration of COVID-19 rally effects may be another point of difference between those on the left vs. right. In the United States, for example, Republicans tend to be more concerned with the impact of COVID-19 on the economy (Samore et al., 2021). Shino and Binder’s (2020) sample of Florida voters showed 83% of Democrats considered COVID-19 a greater public health (vs. economic) threat, compared to 52% of Republicans. Analyses of Republican legislators’ Twitter content similarly reveal a greater focus on economic recovery and assisting small businesses compared to Democrats, who tend to focus more on support for unemployment, housing, and loss of life (Guntuku et al., 2021). In New Zealand, the state of the economy has been a top political issue in recent elections (Vowles et al., 2017; Elder et al., 2021). However, during the October 2020 election, National voters (i.e., the main center-right party) prioritized the economy more than Labour voters (i.e., the main center-left party), viewing it as more important than even COVID-19 (Elder et al., 2021).

These political differences are perhaps unsurprising, given the various epistemic, existential, and relational motivations underlying political ideology (Jost et al., 2009; see also Collins et al., 2021). Indeed, Ruisch et al. (2021) found differences in empathy could (partly) explain the link between ideology and concern about COVID-19. Political differences may also emerge from partisans’ motivation to view their party’s position on political issues positively and consistent with their own view (Bolsen et al., 2014; Leeper and Slothuus, 2014). For example, Goldstein and Wiedemann (2022) identified greater compliance with stay-at-home orders within Republican-leaning counties that had Republican rather than Democrat governors. Further, Jørgensen et al. (2021) found voting for the government to be a strong predictor of support for the government COVID-19 response across eight Western democracies. This suggests that partisans who typically oppose the governing party may be less motivated to listen to, and maintain compliance with, the wishes of that party over time. As such, satisfaction with the governing party may decrease, and concerns about other issues such as the impact of lockdowns on the economy may increase quicker over time. Thus, COVID-19 restrictions may impact political attitudes differently due to both how partisans view the restrictions and their outcomes, and partisans’ receptivity to messaging from the party in power.

### Overview and hypotheses

**Figure 1:**
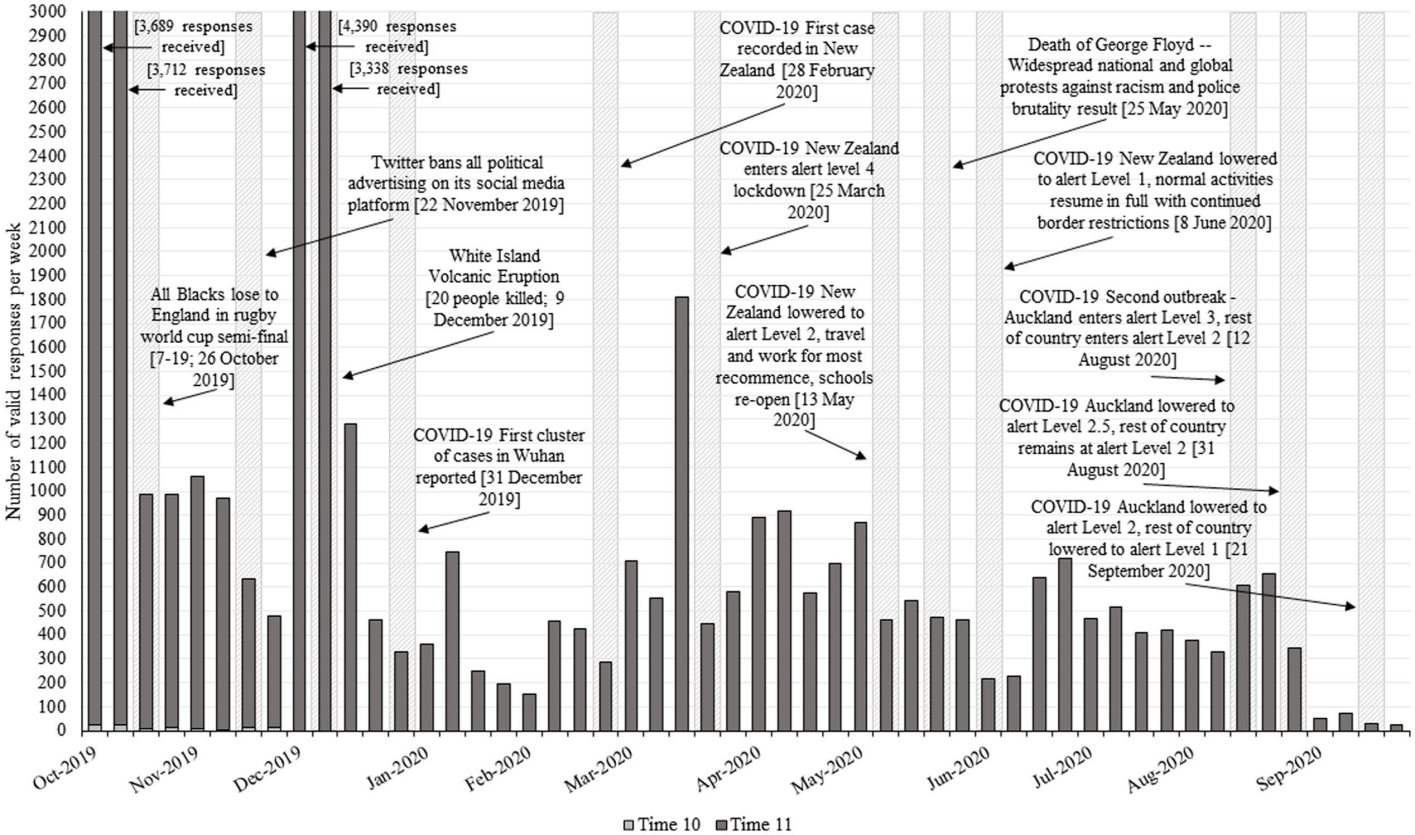
Timeline of responses received during data collection for Time 11, along with COVID-19 and other notable events occurring in New Zealand.

Here, we used a large national probability sample of New Zealand adults from the New Zealand Attitudes and Values Study (NZAVS) to examine trends in key political attitudes in the days and months prior to, and following, New Zealand’s first nationwide COVID-19 lockdown. Announced on March 23, 2020, New Zealanders first learned the government was willing to take sudden and drastic action to reduce the spread of COVID-19 (Bloomfield, 2020). Given the unitary system in New Zealand, these restrictions applied equally to approximately 5 million New Zealanders, regardless of region. Figure 1 provides a timeline of data collection before and after this announcement, along with notable COVID-19 events. The lockdown commenced with “Alert Level 4” of New Zealand’s initial COVID-19 protection framework on March 25. This required non-essential businesses, education and workplaces to close, and everyone to remain in household bubbles, leaving only for exercise or for essential travel (with some exceptions including single person households and shared childcare arrangements). After approximately 1 month, the country shifted to “Alert Level 3,” which also required people to stay home but enabled them to go to work if they could not do so remotely. Some children were also allowed to return to schools at Alert Level 3. “Alert Level 2” was gradually introduced from May 13, allowing restaurants, shops, and eventually bars to reopen, but with limits on gathering sizes. Finally, the country moved to “Alert Level 1” from June 8, which saw remaining restrictions dropped and resembled life before COVID-19, but with the national border remaining closed.^1^ As the NZAVS sampled participants between approximately October 2019 and October 2020, our analyses span the approximately 5 months prior to the lockdown announcement, the duration of the initial lockdown restrictions (approximately 77 days) and the return to normal routines.

We examined changes in several political attitudes, including support for the Labour (major governing party) and National (major opposition party) parties, satisfaction with the government, trust in politicians and science, political efficacy and satisfaction with national wellbeing (the economy, business, and social conditions). By taking this broad approach, our analyses provide comprehensive information about the types of political attitudes that may have changed over time (i.e., attitudes assessing the government, as well as attitudes which may be more relevant to pandemic fatigue, such as national wellbeing). We expect to replicate Sibley et al.’s (2021) general findings by identifying an increase in government satisfaction, as well as trust in politicians and science, immediately following the lockdown announcement. That is, we expected to identify a rally effect in these political attitudes. We also expected that, after the initial increase, these attitudes would generally decline over time.

Notably, Sibley et al. (2020) found that New Zealanders’ perceptions of national wellbeing were largely unchanged in the first 18 days of lockdown, compared to pre-pandemic. As economic concerns appear to increase as the pandemic persisted (e.g., Naumann et al., 2020), we expected that perceptions of national wellbeing would gradually (rather than suddenly) decrease following the lockdown announcement. That said, differences between Labour (center-left) and National (center-right) voters should emerge. In short, we expected the rallying effects in government satisfaction and support would wear off more quickly for National voters who should be less motivated to support the governing Labour party over time. We also expected National voters to become more concerned than Labour voters about the economy, perhaps compounding their weakening support for the government.

Given that COVID-19 lockdowns required actions from the public (e.g., stay home and follow public health mandates) to reduce the impacts of the virus, we also assessed perceptions of political efficacy over time. Prior research demonstrates that New Zealanders’ levels of political efficacy were higher during the lockdown, compared to before (Milfont et al., 2022). However, we hypothesized here that political efficacy would increase gradually (rather than suddenly) after the lockdown announcement, as New Zealanders received feedback that their compliance with the mandates was effective. For example, during this time, Prime Minister Jacinda Ardern fronted daily press conferences where she both thanked New Zealanders for their compliance (Montiel et al., 2021), and remarked that “You are breaking the chain of transmission” (Jacinda Ardern, 9 April 2020) as COVID-19 cases dropped, fostering a sense of collective agency (Vignoles et al., 2021). Because everyone was required to comply with lockdown mandates, these trends might be similar across voter groups. However, center-right National voters may have been less receptive to this out-party messaging, thereby attenuating (or mitigating entirely) the increase in efficacy among this group.

Finally, we also investigate the potential consequences of these rally effects by examining this set of political attitudes as predictors of compliance with Ministry of Health COVID-19 guidelines in 2021. We generally expected support for the Labour party, satisfaction with the government, trust, efficacy, and satisfaction with national wellbeing indicators to predict higher odds of compliance. However, we expected institutional trust and government satisfaction to be the best predictors of compliance, given their frequent association with measures of COVID-19 directive compliance in the literature (e.g., Bargain and Aminjonov, 2020; Wright et al., 2021). Although we did not have formal hypotheses for comparisons between voters, we were broadly interested in whether, for example, concern about the economy and satisfaction with the government might have a larger impact on compliance among National (vs. Labour) voters. In any case, this analysis allowed us to evaluate how consequential the rally effect and time-course of political attitudes may have been for compliance (which we were unable to assess during the 2020 data collection wave).

## Materials and methods

### Sampling procedure

Data come from Time 11 (2020) and Time 12 (2021) of the NZAVS, a national longitudinal probability sample. Time 11 data were used to assess change in political attitudes pre- and post-lockdown announcement (during 2020), while Time 12 data were used to assess the effect of political attitudes on Ministry of Health guideline compliance (as this variable was only available from Time 12). The full Time 11 (2019) NZAVS contained responses from 42,684 participants. Of these, 36,522 were retained from one or more previous wave of the study, originally sampled from the New Zealand electoral roll. This contains the details of New Zealanders aged 18 and over and who are enrolled to vote, where enrolment is compulsory barring exceptions concerning privacy. A Facebook advertisement also targeted people living in New Zealand aged 18 and over from 4th April to 4th July and 18th August to 4th September 2020 in order to ensure the sample size was maximized during the COVID-19 lockdown and recovery phase. This advertising led to an additional 4,734 participants entering the study and a further 1,372 previously ‘lost’ participants re-joining the study. The Time 12 (2020) NZAVS contained responses from 38,551 participants (38,345 retained from one or more previous wave). In both Time 11 and Time 12, participants were provided a link to complete the questionnaire online, but were otherwise sent a physical survey to complete if they had not completed it online or did not provide an email address.

### Participants

Participants in the Time 11 wave had a mean age of 51.56 (*SD* = 13.88), and 64% were women. Regarding ethnicity, 93% identified as New Zealand European, 10% as Māori (Indigenous), 4% as Asian, 3% as Pacific, and 3% as some other ethnicity (percentages do not sum to 100 as people could report more than one ethnic group).

Participants in the Time 12 wave had a mean age of 52.96 (*SD* = 13.7), and 64% were women. Regarding ethnicity, 92% identified as New Zealand European, 9% as Māori, 4% as Asian, 2% as Pacific, and 4% as some other ethnicity.

### Measures

In Time 11 and Time 12, participants stated the party of whom they intended to give (or had given) their party vote at the 2020 election, with 15,211 participants (36.5% of valid responses in Time 11) indicating the Labour party and 9,843 (23.6%) indicating National party (remaining responses included those voting for other minor parties, and those who were unsure or had no intention to vote). They also provided their level of support for the governing, center-left Labour party and opposition, center-right National party from 1 (*Strongly oppose*) to 7 (*Strongly support*), and their satisfaction with “the performance of the current New Zealand government,” “the economic situation in New Zealand,” “business in New Zealand,” and “the social conditions in New Zealand” from 0 (*Completely dissatisfied*) to 10 (*Completely satisfied*).

Trust in politicians was assessed with the single item, “Politicians in New Zealand can generally be trusted” from 1 (*Strongly disagree*) to 7 (*Strongly agree*). Trust in science was assessed with the items “Our society places too much emphasis on science” (reverse-scored) and “I have a high degree of confidence in the scientific community” (r = 0.50), rated from 1 (*Strongly disagree*) to 7 (*Strongly agree*).

Political efficacy was assessed as the average of three items (α = 0.61): “By taking an active part in political and social affairs we, the people, can control world events,” “The average citizen can have an influence on government decisions,” and “With enough effort we can wipe out political corruption.” Items were rated from 1 (*Strongly disagree*) to 7 (*Strongly agree*) and were based on Paulhus and Van Selst’s (1990) socio-political control scale.

Finally, in Time 12, compliance with COVID-19 mandates was assessed with the item “I am willing to strictly follow any and all guidelines provided by the Ministry of Health for managing COVID-19 in New Zealand.” This item was rated from 1 (*Strongly disagree*) to 7 (*Strongly agree*).

### Analytic strategy

To assess which political attitudes changed, and for how long, following the COVID-19 lockdown announcement, we conducted a regression discontinuity analysis with maximum likelihood estimation on the full Time 11 sample, and then among Labour and National party voters separately. This method allows us to assess whether a discontinuity (or “step”) in political attitudes occurred among Time 11 responses received in the days immediately following, compared to immediately before, the lockdown announcement. In other words, it is a *between-persons* analysis that detects trends in attitudes over time both before and after the lockdown announcement, based on responses received by independent samples of participants who responded before and after the announcement.

We used the day in which each participant completed the survey to predict each political attitude, with the day-by-day series centered on 23 March 2020 (the day New Zealand’s Prime Minister and leader of the Labour Party, Jacinda Ardern, announced the country would enter a full “Level 4” lockdown on 25th March), such that 0 represented 23 March 2020. A discontinuity, or “d,” variable was coded 0 for values prior to zero/23 March, and 1 for values equal to or greater than zero/23 March. Thus, the d value represents the difference in the outcome variable or political attitude between responses received before and after the announcement. By including linear, quadratic, and cubed values of day of survey completion, as well as interaction terms between ‘d’ and each of these values, the model accounts for both non-linear trends in political attitudes over time and a change in the trend over time after the lockdown announcement. This allows us to detect a “bottoming out” or peaking of each political attitude as participants respond to the lockdown, as well as any subsequent reversion back to “baseline” levels. Tables 1–3 display the coefficients for these models. Although the models were conducted on all responses received over a one-year period, we plotted the data over the 150 days prior to (approximately 5 months), and 150 days following, the lockdown announcement, as sample sizes per week were notably smaller in the final month of data collection for the Time 11 wave (i.e., during September 2020).

**Table 1:** Regression coefficients estimating trajectories of support for the Labour party, National party, and satisfaction with the government, before and after the lockdown announcement.

	Overall model				Labour voters				National voters			
	*B*	*SE*	*T*	*p*	*B*	*SE*	*T*	*p*	*B*	*SE*	*T*	*p*
**Support for the Labour party**												
Day	0.335	0.235	1.423	0.155	−0.001	0.215	−0.006	0.995	0.645	0.411	1.570	0.116
Day^2^	0.026	0.302	0.086	0.932	−0.084	0.277	−0.301	0.763	0.535	0.524	1.022	0.307
Day^3^	−0.072	0.106	−0.675	0.500	−0.042	0.098	−0.427	0.670	0.148	0.185	0.800	0.424
d	0.260^*^	0.075	3.491	<0.001	0.042	0.063	0.663	0.507	0.333^*^	0.138	2.417	0.016
d × Day	0.483	0.380	1.271	0.204	0.340	0.325	1.043	0.297	−2.264^*^	0.711	−3.185	0.001
d × Day^2^	−1.547^*^	0.495	−3.127	0.002	−0.226	0.427	−0.530	0.596	1.054	0.925	1.139	0.255
d × Day^3^	0.703^*^	0.181	3.875	<0.001	0.140	0.157	0.891	0.373	−0.638	0.340	−1.878	0.060
**Support for the National party**												
Day	−0.392	0.252	−1.558	0.119	−0.045	0.312	−0.144	0.886	−0.068	0.305	−0.221	0.825
Day^2^	−0.012	0.322	−0.037	0.971	−0.081	0.403	−0.202	0.840	−0.081	0.389	−0.209	0.834
Day^3^	0.130	0.114	1.142	0.253	−0.007	0.143	−0.048	0.962	−0.016	0.137	−0.116	0.908
d	0.100	0.080	1.253	0.210	0.169	0.092	1.851	0.064	−0.066	0.102	−0.644	0.519
d × Day	−3.109^*^	0.406	−7.656	<0.001	−2.352^*^	0.473	−4.977	<0.001	−0.081	0.528	−0.153	0.879
d × Day^2^	5.097^*^	0.529	9.639	<0.001	3.169^*^	0.620	5.110	<0.001	0.444	0.688	0.646	0.518
d × Day^3^	−2.042^*^	0.194	−10.530	<0.001	−1.091^*^	0.228	−4.784	<0.001	−0.151	0.252	−0.599	0.549
**Satisfaction with the government**												
Day	0.611	0.359	1.701	0.089	1.245^*^	0.382	3.264	0.001	−0.437	0.681	−0.641	0.522
Day^2^	0.092	0.461	0.199	0.842	1.131^*^	0.493	2.295	0.022	−0.866	0.869	−0.997	0.319
Day^3^	−0.024	0.163	−0.150	0.880	0.348^*^	0.174	1.993	0.046	−0.210	0.307	−0.685	0.493
d	1.144^*^	0.114	10.034	<0.001	0.701^*^	0.112	6.254	<0.001	1.733^*^	0.229	7.557	<0.001
d × Day	0.227	0.581	0.390	0.696	−0.128	0.578	−0.221	0.825	−5.329^*^	1.182	−4.509	<0.001
d × Day^2^	−2.289^*^	0.756	−3.029	0.002	−2.867^*^	0.758	−3.784	<0.001	6.802^*^	1.539	4.421	<0.001
d × Day^3^	0.953^*^	0.277	3.442	0.001	0.287	0.278	1.030	0.303	−1.677^*^	0.565	−2.969	0.003

*N*_(support for the Labour party)_ = 41,850 (15,120 Labour voters, 9,754 National voters). *N*_(support for the National party)_ = 41,863 (15,112 Labour voters, 9,776 National voters). *N*_(satisfaction with the government)_ = 42,514 (15,154 Labour voters, 9,809 National voters).

* *p* < 0.05.

**Table 2:** Regression coefficients estimating trajectories of trust in science, politicians, and political efficacy, before and after the lockdown announcement.

	Overall model				Labour voters				National voters			
	*B*	*SE*	*T*	*p*	*B*	*SE*	*T*	*p*	*B*	*SE*	*T*	*p*
**Trust in science**												
Day	0.027	0.183	0.148	0.883	0.121	0.296	0.409	0.683	0.866*	0.370	2.339	0.019
Day^2^	−0.270	0.239	−1.131	0.258	−0.198	0.388	−0.511	0.610	0.805	0.476	1.690	0.091
Day^3^	−0.211^*^	0.085	−2.476	0.013	−0.196	0.139	−1.416	0.157	0.169	0.169	1.002	0.317
d	0.100	0.054	1.867	0.062	0.026	0.082	0.321	0.748	−0.067	0.121	−0.557	0578
d × Day	1.611^*^	0.279	5.763	<0.001	1.752^*^	0.433	4.049	<0.001	0.628	0.627	1.001	0.317
d × Day^2^	−2.459^*^	0.366	−6.715	<0.001	−2.903^*^	0.571	−5.087	<0.001	−3.262^*^	0.818	−3.988	<0.001
d × Day^3^	1.304^*^	0.134	9.709	<0.001	1.455^*^	0.210	6.930	<0.001	0.776^*^	0.300	2.582	0.010
**Trust in politicians**												
Day	−0.731^*^	0.191	−3.822	<0.001	−0.181	0.314	−0.576	0.565	−0.691	0.438	−1.577	0.115
Day^2^	−0.882^*^	0.245	−3.596	<0.001	−0.208	0.405	−0.513	0.608	−0.819	0.558	−1.467	0.142
Day^3^	−0.273^*^	0.087	−3.157	0.002	−0.057	0.144	−0.400	0.689	−0.236	0.197	−1.196	0.232
d	0.439^*^	0.061	7.241	<0.001	0.361^*^	0.092	3.927	<0.001	0.298^*^	0.147	2.025	0.043
d × Day	0.190	0.309	0.614	0.539	−0.150	0.475	−0.317	0.751	0.029	0.757	0.038	0.969
d × Day^2^	1.122^*^	0.402	2.791	0.005	−0.066	0.622	−0.107	0.915	1.628	0.986	1.652	0.099
d × Day^3^	0.250	0.147	1.698	0.090	0.268	0.229	1.171	0.242	−0.077	0.362	−0.214	0.831
**Political efficacy**												
Day	−0.189	0.162	−1.167	0.244	0.119	0.263	0.455	0.649	0.047	0.353	0.134	0.894
Day^2^	−0.408^*^	0.207	−1.968	0.049	0.131	0.339	0.386	0.699	−0.155	0.450	−0.344	0.731
Day^3^	−0.189^*^	0.073	−2.586	0.010	0.019	0.120	0.159	0.874	−0.098	0.159	−0.614	0.539
d	0.081	0.051	1.577	0.113	0.025	0.077	0.327	0.744	−0.039	0.119	−0.329	0.742
d × Day	1.496^*^	0.261	5.724	<0.001	1.177^*^	0.398	2.956	0.003	0.798	0.612	1.303	0.193
d × Day^2^	−1.669^*^	0.340	−4.908	<0.001	−2.050	0.522	−3.927	<0.001	−1.051	0.797	−1.318	0.188
d × Day^3^	0.996^*^	0.125	8.000	<0.001	0.689	0.192	3.592	<0.001	0.527	0.293	1.801	0.072

*N*_(Trust in science)_ = 42,309 (15,182 Labour voters, 9,831 National voters)*. N*_(Trust in politicians)_ = 41,831 (14,927 Labour voters, 9,663 National voters). *N*_(Political efficacy)_ = 42,663 (15,207 Labour voters, 9,842 National voters).

* *p* < 0.05.

**Table 3:** Regression coefficients estimating trajectories of satisfaction with the economy, social conditions, and business, before and after the lockdown announcement.

	Overall model				Labour voters				National voters			
	*B*	*SE*	*T*	*p*	*B*	*SE*	*T*	*p*	*B*	*SE*	*T*	*p*
**Satisfaction with the economy**												
Day	−1.298^*^	0.287	−4.520	<0.001	−1.240^*^	0.469	−2.641	0.008	−0.628	0.647	−0.972	0.331
Day^2^	−1.282^*^	0.368	−3.481	<0.001	−1.284^*^	0.606	−1.118	0.034	−0.304	0.825	−0.369	0.712
Day^3^	−0.324^*^	0.130	−2.493	0.013	−0.370	0.215	−1.724	0.085	0.085	0.291	0.293	0.770
d	0.384^*^	0.091	4.202	<0.001	0.398^*^	0.138	2.881	0.004	0.355	0.218	1.624	0.104
d × Day	−1.075^*^	0.465	−2.312	0.021	−0.323	0.712	−0.453	0.650	−5.087^*^	1.125	−4.521	<0.001
d × Day^2^	3.899^*^	0.605	6.448	<0.001	2.999^*^	0.933	3.213	0.001	6.817^*^	1.464	4.656	<0.001
d × Day^3^	−0.528^*^	0.221	−2.386	0.017	−0.174	0.343	−0.509	0.611	−2.285^*^	0.537	−4.252	<0.001
**Satisfaction with social conditions**												
Day	−0.748^*^	0.286	−2.613	0.009	−0.463	0.491	−0.943	0.345	−0.502	0.605	−0.831	0.406
Day^2^	−0.744^*^	0.367	−2.028	0.043	−0.384	0.634	−0.606	0.545	−0.401	0.771	−0.520	0.603
Day^3^	−0.126	0.130	−0.971	0.332	0.003	0.224	0.012	0.990	−0.049	0.272	−0.179	0.858
d	0.285^*^	0.091	3.134	0.002	0.243	0.144	1.686	0.092	0.422^*^	0.203	2.073	0.038
d × Day	−2.415^*^	0.463	−5.214	<0.001	−1.791^*^	0.744	−2.408	0.016	−2.050	1.048	−1.956	0.051
d × Day^2^	5.175^*^	0.603	8.586	<0.001	3.708^*^	0.975	3.804	<0.001	3.714^*^	1.365	2.721	0.007
d × Day^3^	−1.508^*^	0.221	−6.829	<0.001	−1.283^*^	0.358	−3.582	<0.001	−1.160^*^	0.501	−2.315	0.021
**Satisfaction with business**												
Day	−1.677^*^	0.255	−6.567	<0.001	−1.821^*^	0.417	−4.364	<0.001	−0.822	0.593	−1.387	0.165
Day^2^	−1.630^*^	0.328	−4.976	<0.001	−1.764^*^	0.539	−3.274	0.001	−0.584	0.756	−0.773	0.440
Day^3^	−0.383^*^	0.116	−3.311	0.001	−0.456^*^	0.191	−2.392	0.017	0.011	0.267	0.041	0.967
d	0.108	0.081	1.333	0.182	0.216	0.122	1.763	0.078	0.125	0.199	0.629	0.529
d × Day	−0.092	0.413	−0.223	0.824	0.110	0.632	0.173	0.862	−3.218^*^	1.027	−3.133	0.002
d × Day^2^	4.189^*^	0.537	7.799	<0.001	4.313^*^	0.829	5.205	<0.001	5.759^*^	1.337	4.308	<0.001
d × Day^3^	−0.566^*^	0.197	−2.875	0.004	−0.472	0.304	−1.550	0.121	−1.922^*^	0.491	−3.916	<0.001

*N*_(Economy)_ = 42,393 (15,093 Labour voters, 9,800 National voters). *N*_(Social conditions)_ = 41,831 (15,138 Labour voters, 9,796 National voters). *N*_(Business)_ = 42,354 (15,103 Labour voters, 9,799 National voters).

* *p* < 0.05.

Following our analysis of change in political attitudes before and after the March 2020 lockdown, we conducted a regression analysis using the Time 12 (2021) sample to determine which political attitudes contributed to peoples’ willingness to follow New Zealand Ministry of Health’s guidelines for reducing the spread of COVID-19 (e.g., social distancing). Although responses could vary from 1 to 7, most participants expressed the maximum (7; 65.1%) or near maximum (6; 19.7%) levels of agreement with the item. In other words, the variable was highly skewed with the clear majority selecting these two response options. Although we have a large sample size, this represented severe skewness in the variable. Thus, instead of analyzing the data with a standard linear regression or alternative models that may help account for the skewness, we opted to dichotomize responses into those who expressed agreement with the item (ratings of 5–7; scored 1) and those who were at the mid-point or disagreed with the item (ratings of 1–4; scored 0) and used a logistic regression to predict these responses. Dichotomizing the variable in this case provided both practical utility (i.e., it is practical to know who will comply and why, and who is unsure or will not) and greater confidence in both the model assumptions being met and regression effects. For ease of interpreting the relative effects of each predictor variable, we also rescaled all variables to range from 0 to 1 for this analysis. Regression estimates and odds ratios therefore reflect the difference between the highest and lowest scores on each predictor.

## Results

### Time course of political attitudes before and after the march 2020 lockdown

#### Attitudes toward government

**Figure 2:**
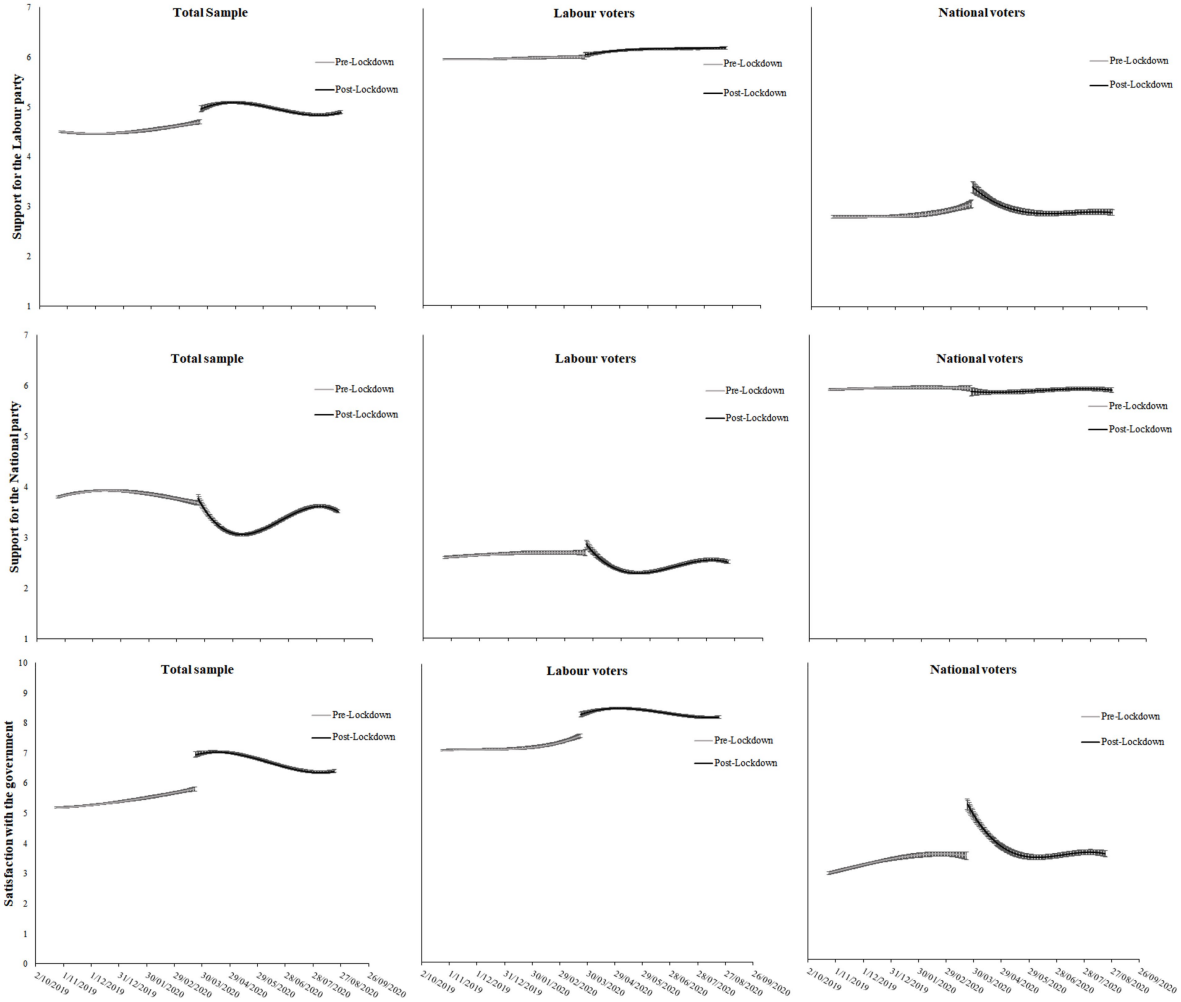
Trajectories of support for the Labour party, National party and satisfaction with the government pre- and post-Lockdown. Regression coefficients for the trajectories can be found in Table 1.

Support for the governing center-left (Labour) party and satisfaction with the government each increased immediately following the Level 4 lockdown announcement (see d coefficients in Table 1; Figure 2 for the overall trend). Although there was a gradual decrease in support and satisfaction, both attitudes remained elevated above pre-lockdown levels of the duration examined (approximately 5 months). There were, however, notable differences when examining Labour and National voters separately. Labour voters did not change their levels of support for the Labour party following the lockdown announcement, nor during the next 5 months (indicated by the non-significant d coefficient and interaction terms in Table 1). This was likely due to ceiling effects in their levels of support. By contrast, National voters saw an immediate increase in support for the Labour party (d coefficient = 0.33, *p* = 0.016; Table 1), returning to pre-lockdown levels after approximately 29 days. Both Labour and National voters also saw an immediate increase in their satisfaction with the government (d coefficient = 0.70 and 1.73 respectively, *p*s < 0.001; Table 1). Whereas Labour voters maintained this increase in satisfaction throughout the 5-month post-lockdown period, National voters returned to pre-lockdown levels after about 60 days. Support for the opposition center-right National party is also displayed for comparison, with support decreasing among the total sample and Labour voters specifically, and not recovering during the period examined (although it remained very high among National voters).

Overall, National voters’ initially lower levels of support for the Labour party and satisfaction with the government resulted in a bigger boost in these attitudes relative to Labour voters immediately following lockdown. However, these boosts, or rallying effects, dissipated after 60 days. As such, National voters tended to rally around the government for a shorter period following the announcement of the Level 4 lockdown.

#### Trust and political efficacy

**Figure 3:**
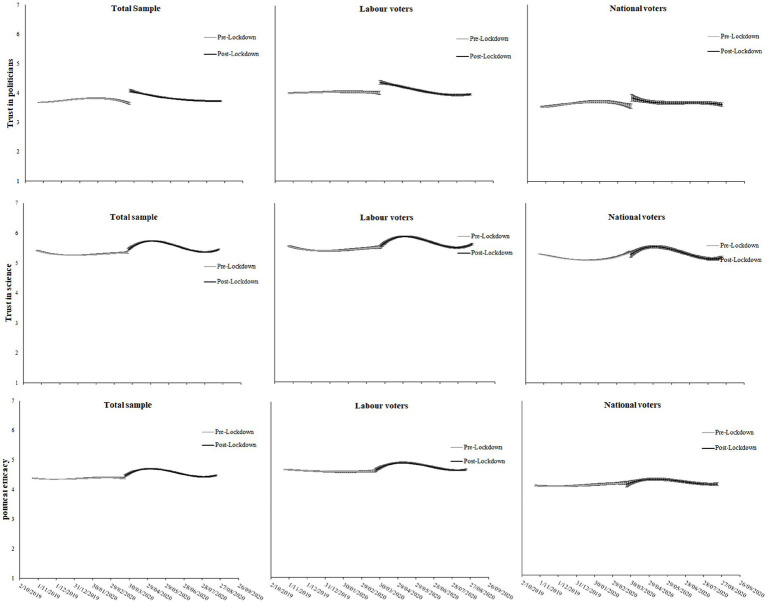
Trajectories of trust in politicians, trust in science, and political efficacy pre- and post-Lockdown. Regression coefficients for the trajectories can be found in Table 2 (note the downward trend in trust in politicians among Labour voters post-Lockdown is not significant).

Figure 3 displays the trajectories of trust in politicians, trust in science, and political efficacy, with regression results displayed in Table 2. Increases in trust in both politicians and science, as well as political efficacy, were also observed after the Level 4 lockdown announcement. Trust in politicians increased immediately following the announcement, for the total sample (d coefficient = 0.44, *p* < 0.001) and Labour (d coefficient = 0.36, *p* < 0.001) and National voters (d coefficient = 0.30, *p* = 0.043) separately. Moreover, these increased levels of trust remained steady throughout the 5 months following the announcement (note that the apparent decreasing trend in trust in politicians for Labour voters in Figure 3 is non-significant; see Table 2). Trust in science also increased after the lockdown announcement, but not immediately. Instead, trust in science grew gradually over time for both Labour and National voters. For both Labour and National voters, trust in science tended back toward pre-lockdown levels toward the end of the 5-month period, reaching pre-lockdown levels slightly sooner for National voters (around 3-months on) compared to Labour voters (see Figure 3). Finally, political efficacy increased in the total sample, and among Labour voters (but not National voters; see non-significant effects of d x day, day^2^ and day^3^ in Table 2), gradually following the lockdown announcement.

#### National wellbeing

**Figure 4:**
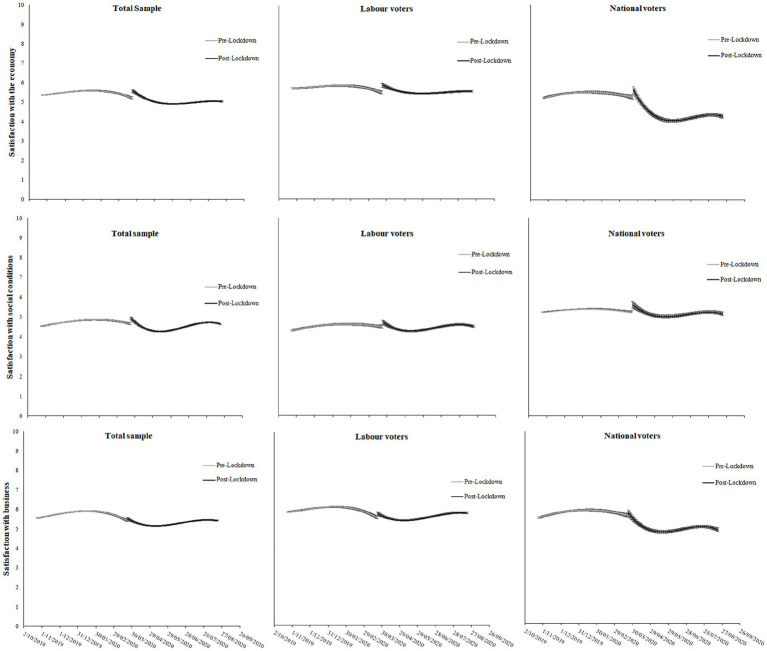
Trajectories of satisfaction with the economy, social conditions, and business, pre- and post-Lockdown. Regression coefficients for the trajectories can be found in Table 3.

Figure 4 displays the trends in different indices of national wellbeing before and after the lockdown, with corresponding regression coefficients presented in Table 3. Here, analysis of the total sample indicates relatively subdued changes in each measure following the lockdown. Satisfaction with the economy was on a small downward trend prior to the lockdown announcement, yet increased slightly immediately after the announcement (d coefficient = 0.38, *p* < 0.001), before decreasing below pre-lockdown levels. A similar trend was observed for satisfaction with social conditions (d coefficient = 0.29, *p* = 0.002) and business, although there was no change in satisfaction immediately following the lockdown announcement for business (d coefficient = 0.11, *p* = 0.182). In each case, satisfaction by the end of the 5-month post-lockdown period was about where it was just before the lockdown.

Differences between Labour and National voters were also evident. Labour voters’ satisfaction with the economy, business, and social conditions was generally similar to the total sample over time. By contrast, National voters’ satisfaction with the economy and business dropped drastically over time (see Figure 4). Their satisfaction with social conditions did not significantly change immediately, but instead, very subtly decreased before increasing again. Satisfaction with the economy bottomed out approximately 66 days after the lockdown announcement, at a rating of 4.05 (compared to 5.27 just before the announcement), with a slow recovery in satisfaction seen thereafter, but which remained well below pre-lockdown levels. Satisfaction with business reached its lowest point approximately 55 days following the lockdown announcement, before also recovering slightly. Notably, the timing of these trends aligns roughly with when restrictions were lifted in New Zealand, as the country took more significant steps toward normality on 11 May (47 days post-lockdown), and relaxed restrictions on social gatherings from 25 May (61 days post-lockdown).

### Which political attitudes matter most in predicting compliance?

Table 4 displays correlations between our focal measures at Time 12 (2021), and means and standard deviations at Time 11 (2020) and Time 12. Labour party support (*r = 0*.39), satisfaction with the government (*r = 0*.39), and trust in politicians (*r = 0*.30) had the highest correlations with Ministry of Health guideline compliance when measured on the original (1–7) scale. Table 5 displays logistic regression estimates for each political attitude predicting COVID-19 guideline compliance (where 1 indicates agreement to comply, and 0 indicates uncertainty or disagreement with complying) for the total sample, as well as separately for Labour (governing) and National (opposition) voters using the full Time 12 wave of the NZAVS.^2^ In the total sample, all political attitudes predicted the odds of following guidelines, with trust in science having the largest positive effect (whereby higher levels of trust predicted greater odds of compliance), followed by satisfaction with the government. Satisfaction with different aspects of national wellbeing had comparatively smaller effects, where greater satisfaction with the economy and business, but less satisfaction with social conditions, was associated with greater odds of compliance. Differences were, however, again present between Labour and National voters when conducting the regression among these groups separately. For Labour voters, trust in science had by far the largest effect on compliance, as the odds of compliance for those expressing maximum levels of trust were 15.7 times higher than the odds of those with the lowest levels of trust. Satisfaction with the government, followed by satisfaction with business, were the second and third most influential political attitudes (respectively), with the odds those with the highest levels of satisfaction about 4.6 and 4.1 times the odds of those with the lowest levels of satisfaction.

**Table 4:** Correlations between focal measures at Time 12 (2021) and means and standard deviations at Time 11 (2020) and 12.

	1	2	3	4	5	6	7	8	9	10
1. Labour party support	-									
2. National party support	−0.574^*^	-								
3. Trust in politicians	0.391^*^	−0.082^*^	-							
4. Trust in science	0.205^*^	−0.114^*^	0.258^*^	-						
5. Political efficacy	0.332^*^	−0.215^*^	0.406^*^	0.261^*^	-					
6. Satisfaction with the government	0.779^*^	−0.454^*^	0.462^*^	0.195^*^	0.329^*^	-				
7. Satisfaction with the economy	0.307^*^	−0.040^*^	0.353^*^	0.142^*^	0.202^*^	0.485^*^	-			
8. Satisfaction with social conditions	−0.020^*^	0.240^*^	0.183^*^	−0.036^*^	0.021^*^	0.188^*^	0.433^*^	-		
9. Satisfaction with business	0.210^*^	0.042^*^	0.334^*^	0.155^*^	0.193^*^	0.378^*^	0.526^*^	0.385^*^	-	
10. Ministry of health compliance	0.385^*^	−0.136^*^	0.300^*^	0.274^*^	0.245^*^	0.389^*^	0.217^*^	0.009	0.179^*^	-
Time 11 (2020) Mean (SD)	4.66 (1.80)	3.67 (1.93)	3.76 (1.45)	5.43 (1.28)	4.45 (1.24)	5.75 (2.83)	5.31 (2.21)	4.60 (2.20)	5.56 (1.96)	-
Time 12 (2021) Mean (SD)	4.80 (1.82)	3.51 (1.83)	4.04 (1.50)	5.64 (1.25)	4.41 (1.23)	5.90 (2.80)	5.38 (2.25)	4.58 (2.19)	5.80 (1.93)	6.34 (1.22)

Ministry of Health compliance presented on the original (1–7) scale.

* *p* < 0.01.

**Table 5:** Logistic regression of political attitudes predicting compliance with Ministry of Health COVID-19 guidelines.

	Total sample					Labour voters					National voters				
	*b*	*se*	*OR*	*OR 95% CI*	*p*	*b*	*se*	*OR*	*OR 95% CI*	*p*	*b*	*se*	*OR*	*OR 95% CI*	*p*
Gender	−0.610	0.046	0.543	0.497, 0.594	<0.001	−0.701	0.105	0.496	0.404, 0.610	<0.001	−0.596	0.084	0.551	0.467, 0.649	<0.001
Age	1.308	0.129	3.699	2.872, 4.764	<0.001	1.770	0.282	5.869	3.377, 10.198	<0.001	1.111	0.269	3.037	1.794, 5.140	<0.001
Ethnicity	0.127	0.077	1.136	0.976, 1.321	0.100	−0.034	0.168	0.967	0.695, 1.345	0.842	0.246	0.155	1.279	0.944, 1.733	0.113
Employed	0.127	0.056	1.135	1.017, 1.267	0.024	0.401	0.115	1.494	1.193, 1.870	<0.001	0.023	0.109	1.023	0.826, 1.266	0.836
Labour party support	1.055	0.115	2.871	2.290, 3.599	<0.001	0.705	0.285	2.023	1.157, 3.537	0.013	0.987	0.252	2.684	1.638, 4.399	<0.001
Satisfaction with government	2.043	0.140	7.716	5.866, 10.149	<0.001	1.515	0.297	4.551	2.544, 8.139	<0.001	2.288	0.279	9.856	5.699, 17.044	<0.001
Trust in politicians	1.125	0.112	3.079	2.474, 3.833	<0.001	1.084	0.247	2.957	1.822, 4.800	<0.001	0.518	0.197	1.679	1.140, 2.472	0.009
Trust in science	2.426	0.101	11.314	9.278, 13.798	<0.001	2.753	0.225	15.690	10.102, 24.369	<0.001	2.024	0.189	7.568	5.222, 10.967	<0.001
Political efficacy	0.800	0.117	2.225	1.769, 2.798	<0.001	0.997	0.276	2.710	1.578, 4.654	<0.001	0.939	0.223	2.558	1.654, 3.958	<0.001
Satisfaction with the economy	0.643	0.123	1.902	1.494, 2.421	<0.001	−0.145	0.309	0.865	0.472, 1.584	0.638	0.763	0.219	2.144	1.395, 3.297	0.001
Satisfaction with social conditions	−0.616	0.115	0.540	0.431, 0.677	<0.001	−0.048	0.296	0.953	0.534, 1.702	0.870	−0.804	0.210	0.448	0.297, 0.676	<0.001
Satisfaction with business	0.442	0.133	1.556	1.198, 2.021	0.001	1.422	0.365	4.145	2.026, 8.481	<0.001	0.411	0.227	1.508	0.966, 2.356	0.071

*N*_(Total sample)_ = 35,320*. N*_(Labour voters)_ = 16,287. *N*_(National voters)_ = 6,907.

Among National voters, satisfaction with the government produced the largest effect on compliance, followed by trust in science. For National voters with the maximum level of satisfaction with the government, the odds of compliance were 9.9 times higher than the odds of those with the lowest satisfaction. For trust in science, odds of compliance were 7.6 times higher at the highest levels of trust vs. lowest levels. Despite National voters expressing notable decreases in satisfaction with the economy and business during lockdown, satisfaction with the economy had a comparatively smaller effect on compliance, with an odds ratio of 2.1 between the highest and lowest levels of satisfaction, while satisfaction with business was unassociated with compliance. These differences should also be kept in perspective with the overall extremely high rates of compliance in New Zealand. Specifically, only 8.1% of the total sample were unsure or unwilling to comply with all guidelines from the Ministry of Health. Nevertheless, this percentage was notably higher among National voters (11.6%) compared to Labour voters (2.8%).

## Discussion

Effective pandemic management requires prolonged cooperation among all segments of society. Although rally-around-the-flag effects have been documented in the initial phases of the COVID-19 pandemic (Sibley et al., 2020; Bol et al., 2021), less is known about how long public opinion remains favorable toward political institutions and whether these trends vary by political party vote. In the current study, we (a) analyzed the trajectory of political attitudes before and after New Zealand’s March 2020 COVID-19 lockdown, (b) examined differences in these trends between center-right National and center-left Labour voters, and (c) tested the associations between these political attitudes and participants’ willingness to follow guidelines to reduce the spread of COVID-19.

Our results identified rally-around-the-flag effects such that New Zealanders’ support for the (governing) Labour party and satisfaction with the government increased following the lockdown announcement and remained elevated for the following 5 months. This likely translated to the unprecedented party vote received by the Labour party in the October 2020 national election. Specifically, Labour’s vote share increased by 13 percentage points to 50%, allowing the party to govern alone. Trust in politicians and science similarly increased following the lockdown and remained above baseline levels for a full 5 months, while political efficacy also gradually increased over that time, perhaps due to persistent political messaging from the government that New Zealanders’ efforts to stay at home effectively reduced the spread of COVID-19. By contrast, despite some indications that perceptions of national wellbeing initially increased following the lockdown announcement, satisfaction with the economy, business, and social conditions tended to decrease over time, and remained below baseline for the following 5 months (economy), or recovered toward the end of the period examined (social conditions and business).

Overall, these results provide crucial insights into how, and for how long, citizens’ political attitudes shifted in relation to the COVID-19 pandemic. Specifically, we observed shifts in some attitudes that exceeded 5 months—shifts that are similar to, if not longer than, attitude change generated by other major events such as terror attacks (Geys and Hernæs, 2021; Satherley et al., 2021). Notably, in the case of New Zealand, increased satisfaction and trust in national institutions, as well as an increased sense of political efficacy, persisted for the duration of (and beyond) the lockdown and government mandates, likely contributing to New Zealand’s successful COVID-19 response. Whereas we largely replicate Sibley et al. (2020) by finding immediate boosts in government and institutional satisfaction, we also identified decreases in national wellbeing that generally emerged over time (rather than immediately following the lockdown). This underscores the importance of examining changes over time in response to large scale events such as the COVD-19 pandemic, particularly as conditions change over time.

Our results also revealed that the duration of political attitude change depends on party vote. Whereas center-left Labour voters tended to resemble the overall sample trends, center-right National voters reverted to baseline levels of support for Labour and satisfaction with the government after about 60 days. National voters also did not experience a boost in political efficacy. Rather, they saw sharp decreases in satisfaction with the economy and business, which did not recover during the 5-month post-lockdown period examined. As such, our results suggest that political divisions may increase throughout the pandemic. Indeed, consistent with past research (Elder et al., 2021) and our hypothesis, the economy appears to be of particular concern for center-right voters. These concerns may be buffered, however, by the increased trust in science and politicians also experienced by National voters. Given trust in both politicians and scientists implies a willingness to listen to those institutions (even if one does not fully understand or agree with them), it may be the key attitude that can bridge political divides and foster mass compliance among the public. Consistent with this thesis, high levels of trust in science attenuates the gap in social distancing practices between conservatives and liberals in the United States (Koetke et al., 2021).

Follow-up analyses approximately 1 year later revealed that every political attitude examined here predicted a willingness to follow Ministry of Health COVID-19 guidelines, underscoring their importance to understanding peoples’ behavior during pandemics. However, trust in science and satisfaction with the government had the largest effects on compliance. The order of importance of these effects did, however, differ between Labour and National voters. Whereas trust in science had the largest effect among Labour voters (i.e., voters for the governing party), satisfaction with the government had the largest effect on compliance among National voters. Notably, although National voters exhibited a sizeable dip in their satisfaction with the economy following the 2020 lockdown, economic satisfaction played a relatively smaller role in predicting compliance. As such, while a key motivation for examining partisan differences in political attitude change was borne out (i.e., increased concern about the economy on the right), these concerns may not play a large role when it comes to compliance. Instead, a government response that garners satisfaction on both the left and right (perhaps including how economic impacts may be mitigated) appears to be the more important factor in reducing partisan divisions when managing COVID-19.

### Strengths, limitations, and future directions

Our analyses utilize data from a large national probability survey of New Zealanders collected over a long period of time. This allows us to generate reasonably reliable estimates of how the public’s political attitudes changed in response to lockdown in a country that has had good success in dealing with COVID-19, particularly by eliminating it early on in the pandemic (Baker et al., 2020). Whereas much work on COVID-19 has focused on attitudes and behaviors specifically about COVID-19, our focus on general political attitudes has multiple benefits. First, it meant we could compare attitude trajectories from before to after COVID-19 became a significant issue, thus providing a useful baseline for assessing change in response to the lockdown. Second, it provides a more nuanced understanding of which attitudes change in response to lockdown, and thus greater insight into citizens’ concerns. Finally, we were able to assess which specific factors contribute most to compliance.

Some attitudes, like satisfaction with the government, seem subject to rally effects that exhibit immediate upticks. Conversely, other attitudes that foster public compliance (e.g., institutional trust and political efficacy) seem to develop over time, possibly through effective and informative communication as the pandemic progresses. These are notable distinctions, as trust in science in particular appears to have the largest influence on compliance within the public on the whole. Other attitudes—specifically, concern about the economy—tend to develop over time, rather than respond immediately to lockdowns (e.g., Sibley et al., 2020). Thus, when faced with sudden drastic events such as global pandemics, governments might expect general immediate support from their citizens. This support may, however, wane over time (especially for opposition party voters) and require counteracting by instilling trust in the institutional response and a sense of efficacy among the public.

It is important to recognize that our analysis represents attitude change in response to the New Zealand context. While this highlights how political attitudes can be expected to change in response to a well-managed institutional response, it also means our results may not reflect attitude change in other countries. Nevertheless, it provides a useful comparison to the sizeable international literature on COVID-19 attitudes, and attitude change across nations. Future research could compare political attitude change across countries that pursued various COVID-19 responses in terms of style (for example, countries with even longer lockdowns or who initiated lockdowns later in the pandemic) and success. Similarly, future research could examine whether rally effects are as large or enduring during subsequent lockdown events (for example, New Zealand was placed in another less restrictive lockdown toward the end of the data collection period for this study, and a strict lockdown for a second time in August 2021).

It is also important to bear in mind that our analyses of attitude trajectories are *between*-person analyses based on different participants responding at different times. This may be particularly important when considering differences in attitude trajectories between Labour and National voters, as people’s intended party vote for the 2020 election may have simultaneously changed with their attitudes (indeed, this is likely reflected in the historic election win for Labour in October 2020). In this sense, apparent increased political divisions in response to COVID-19 restrictions may result in part from only the most committed National supporters maintaining their intended vote for the party over time. Nonetheless, the analyses present a continuous snapshot of Labour and National voters’ attitudes over time based on the day-by-day voter base for each party.

Although our predictions for voter differences in attitude change were based on past theory and research on ideology and partisanship, our analyses cannot distinguish between these factors. The findings here may reflect one or both of ideological differences between voters and partisan differences (particularly regarding whether a voter’s preferred party was in power or not). Finally, we were unable to assess COVID-19 attitudes (i.e., compliance) among participants during the first 2020 lockdown and, thus, our analyses of compliance are from data collected after the lockdown and restrictions ended. This was a time in which New Zealanders enjoyed reasonably lengthy periods of restriction-free life, with the tail end of data collection occurring during the nation’s second significant lockdown in response to the Delta outbreak. As such, compliance, and predictors of compliance, may have differed during lockdowns or during other specific periods of the pandemic.

## Conclusion

A key issue facing nations throughout the COVID-19 pandemic has been ensuring compliance with restrictive and unprecedented lockdowns among citizens over extended periods. The current study produced insights into how New Zealanders’ political attitudes changed over time in response to the first March 2020 COVID-19 lockdown, and how these differed among center-right and center-left National and Labour voters, respectively. Overall, New Zealanders exhibited immediate increases in support for the governing Labour party and satisfaction with the government—changes which endured for the 5-month post-lockdown period examined. Trust in politicians and science, as well as political efficacy, also grew gradually from the start of lockdown. Nevertheless, partisan differences in the time course of these attitudes emerged, as National voters experienced a large and growing concern about the economy, and quickly returned to baseline levels of support for, and satisfaction with, the government. Finally, trust in science and satisfaction with the government emerged as the most influential predictors of compliance, although the order of importance was reversed for National voters. Overall, these results highlight immediate rallying effects in citizens’ attitudes toward their government following COVID-19 lockdown. However, trajectories and magnitudes of change in attitudes over time depend on both the attitude examined and left vs. right party vote.

## Data availability statement

The data analyzed in this study is subject to the following licenses/restrictions: The data analyzed here is part of the New Zealand Attitudes and Values Study (NZAVS). Syntax for the analyses is available on the NZAVS website: www.nzavs.auckland.ac.nz. A de-identified dataset containing the variables analyzed in this manuscript is available upon request for the purpose of replicating the analyses reported here. Requests to access these datasets should be directed to CS, c.sibley@auckland.ac.nz.

## Ethics statement

The studies involving human participants were reviewed and approved by the University of Auckland Human Participants Ethics Committee (Reference Number UAHPEC22576). The patients/participants provided their written informed consent to participate in this study.

## Author contributions

NS conceptualized the study, conducted the analyses, interpreted the results, and wrote the draft manuscript. EZ, LG, FB, DO, and CS provided feedback and revisions on the draft manuscript and interpretation of the results. CS acquired the data and funding for the study. All authors contributed to the article and approved the submitted version.

## Funding

The New Zealand Attitudes and Values Study is funded by a grant from the Templeton Religion Trust (TRT-2021-10418).

## Conflict of interest

The authors declare that the research was conducted in the absence of any commercial or financial relationships that could be construed as a potential conflict of interest.

## Publisher’s note

All claims expressed in this article are solely those of the authors and do not necessarily represent those of their affiliated organizations, or those of the publisher, the editors and the reviewers. Any product that may be evaluated in this article, or claim that may be made by its manufacturer, is not guaranteed or endorsed by the publisher.

## Footnotes

^1^See https://covid19.govt.nz/about-our-covid-19-response/history-of-the-covid-19-alert-system/ for full details on the alert level timeline and restrictions at each level during New Zealand’s COVID-19 response.

^2^Although we opted to use logistic regression here, the effects across predictors in terms of significance and direction were consistent with the logistic regression models when running the model as a standard multiple linear regression.
